# Exploring Socioeconomic Differences in Bedtime Behaviours and Sleep Duration in English Preschool Children

**DOI:** 10.1002/icd.1848

**Published:** 2014-02-19

**Authors:** Caroline H D Jones, Helen Ball

**Affiliations:** a Department of Anthropology, Durham UniversityDurham, UK; b Department of Primary Care Health Sciences, University of OxfordOxford, UK; c Wolfson Research Institute for Health and Wellbeing, Durham UniversityDurham, UK

**Keywords:** sleep, preschool children, sleep hygiene, England, mixed methods, anthropology

## Abstract

Children's sleep is critical for optimal health and development; yet sleep duration has decreased in recent decades, and many children do not have adequate sleep. Certain sleep behaviours (‘sleep hygiene’) are commonly recommended, and there is some evidence that they are associated with longer nighttime sleep. Parents of 84 British 3-year-old children were interviewed about their children's sleep and completed five-night/four-day sleep diaries documenting their children's sleep, from which daily sleep duration was estimated. Diaries were validated by actigraphy in a subgroup of children. Sleep hygiene behaviours (regular bedtime, reading at bedtime, falling asleep in bed) were associated with each other, and were more common in the high socioeconomic status compared to the low socioeconomic status group. Parents' reasons for not practicing sleep hygiene included difficulty, inability or inconvenience. Sleep hygiene behaviours were associated with significantly longer child sleep at night but not over 24 h. Longer daytime napping compensated for shorter nighttime sleep in children whose parents did not implement sleep hygiene behaviours. Parents may need to be advised that certain behaviours are associated with longer nighttime sleep and given practical advice on how to implement these behaviours. © 2014 The Authors. *Infant and Child Development* published by John Wiley & Sons, Ltd.

## INTRODUCTION

Children's sleep is critical for an array of health and behaviour outcomes. Poor or insufficient child sleep is associated with obesity (particularly in younger ages) (Chen, Beydoun, & Wang, 2008), insulin resistance (Flint et al., 2007), executive functioning and mental health (Mindell, Owens, & Carskadon, 1999) and, in school children, reduced learning capacity and academic performance (Curcio, Ferrara, & De Gennaro, 2006), poorer neurobehavioural functioning and behaviour regulation (Sadeh, Gruber, & Raviv, 2002, 2003). Hence there is growing interest in understanding the modifiable factors that influence child sleep. Previous studies have revealed substantial variability in sleep patterning and duration even within populations and age groups (Blair et al., 2012; Iglowstein, Jenni, Molinari, & Largo, 2003; Mindell, Sadeh, Wiegand, How, & Goh, 2010; Sadeh, Mindell, Luedtke, & Wiegand, 2009). Results of a recent infant twin study suggest that the environment, which for young children is predominantly the home and their parents, influences sleep duration to a greater extent than does genetics (Fisher, van Jaarsveld, Llewellyn, & Wardle, 2012). According to the transactional model, there are multiple bidirectional pathways linking parenting with child sleep (Sadeh & Anders, 1993).

Despite the importance of child sleep, it is estimated that sleep duration has decreased in recent decades (Iglowstein et al., 2003), with a recent review reporting a decline in children's sleep of an average 0.73 min/year over the last century (Matricciani, Olds, Blunden, Rigney, & Williams, 2012). When parent-reported usual bedtimes, wake times and daily nap durations were used to estimate daily sleep duration in 253 American children aged 3 months to 12 years, it was found that a quarter did not reach the daily duration of sleep recommended by the National Sleep Foundation for their age (Owens, Jones, & Nash, 2011). Addressing our limited understanding of the causes of variation in child sleep duration is therefore of growing importance.

Certain ‘sleep hygiene’ behaviours including regular sleep–wake schedules, sleeping in a quiet dark room, a consistent bedtime routine ending in the bedroom, absence of bedroom electronics and avoidance of caffeine are commonly recommended to improve sleep (National Sleep Foundation, [Bibr b23].; Galland & Mitchell, 2010) and have been empirically linked to better quality and longer sleep in children (Mindell, Meltzer, Carskadon, & Chervin, 2009). However, sleep hygiene has been seldom evaluated in healthy, non-sleep disordered children (Galland & Mitchell, 2010), nor in British children, where the association may vary due to cross-cultural differences in child sleep (Mindell et al., 2010; Worthman & Melby, 2002). Quantitative data that do exist tell us little about the context of sleep hygiene, for example, why parents do and do not use certain practices. Furthermore a recent review highlighted the paucity of research examining the links between child sleep and parenting in socioeconomically diverse populations (Sadeh, Tikotzky, & Scher, 2010), despite known positive associations between markers of socioeconomic position and sleep (Rona, Li, Gulliford, & Chinn, 1998) and parents' use of regular bedtimes and routines for their children (Hale, Berger, LeBourgeois, & Brooks-Gunn, 2009).

Most recommendations focus predominantly on children's nighttime sleep duration (Mindell et al., 2009), with lesser attention on associations between sleep hygiene and daytime sleep or combined daytime and nighttime sleep (Owens et al., 2011; Sadeh et al., 2009). Evidence is mixed regarding whether or not the composition of children's daily sleep, i.e. consolidated at night versus combined daytime and nighttime, is important for associated health and behaviour (see the Implications section of the Discussion). In this study, we included both nighttime and daytime sleep in analyses.

This mixed methods study addresses current gaps in evidence by examining the use of sleep hygiene behaviours, including parents' reasons for using or not using them, and the association with sleep duration, in a socioeconomically diverse population of healthy, non-sleep disordered British preschool children. We examined sleep hygiene behaviours that are commonly recommended to improve children's sleep (Galland & Mitchell, 2010; Mindell & Owens, 2009; National Sleep Foundation, [Bibr b23].) and have been empirically associated with longer child sleep duration. They are as follows: having a regular bedtime (Owens et al., 2011); reading as part of the bedtime routine (Mindell et al., 2009); and falling asleep in bed each night, not on the sofa (Mindell et al., 2009; Sadeh et al., 2009). Conversely, lack of a regular bedtime, absence of a bedtime routine including reading, parental presence while falling asleep and being put in bed asleep rather than awake have been negatively associated with sleep duration in young children (Mindell et al., 2009; Owens et al., 2011; Sadeh et al., 2009). Our aims were to (i) quantitatively examine sleep duration and use of sleep hygiene behaviours in a socioeconomically diverse sample of British preschool children, drawing comparisons between socioeconomic groups; (ii) qualitatively explore the reasons parents do and do not practice sleep hygiene behaviours; and (iii) examine whether British children benefit from sleep hygiene recommendations, which are based on research conducted elsewhere, by assessing associations between sleep hygiene behaviours and sleep duration.

## METHODS

### Participants

Participants were 3-year-old children and their parents, recruited at government-funded nursery schools in Stockton-on-Tees, an economically diverse town in North-East England. Three nursery schools in areas of particularly low socioeconomic status and two in areas of particularly high socioeconomic status were purposively sampled. According to the Index of Multiple Deprivation, these areas were in the 20% most deprived wards nationally and the 10% least deprived wards, respectively (Stockton Borough Council, [Bibr b34].) – we term these the low socioeconomic status (low SES) and high socioeconomic status (high SES) groups. Data were collected from each nursery consecutively. Parents who could complete interviews and diaries in English were invited to participate, excluding rare cases where nursery staff disclosed that families were involved with the police or social services. No children were medically diagnosed as having a sleep disorder.

Parents of 133 children were invited, and 108 participated (recruitment rate 82%; 83% and 81% in the high SES and low SES groups, respectively). Ninety-one parents returned diaries (84%); some were incomplete leaving 84 families (77%) with complete data who were included in analyses (41 in the high SES group and 43 in the low SES group; 46 boys and 38 girls; age range 36–47 months, mean 41 ± 3 months).

Participant characteristics are shown in Table 1, for the whole sample and by socioeconomic group. In the low SES group compared to the high SES group, there were significantly more non-White British families, fewer children living with both parents and younger mean maternal age. Examining the areas in which the high SES and low SES nurseries were located, there were significant differences in weekly household earnings, proportion of residents claiming income benefits, proportion of adults with poor literacy and numeracy, and proportion of adults unemployed, in the directions expected (Jones, 2011; Stockton Borough Council, [Bibr b34].).

**Table 1:** Participant characteristics

Characteristic	Whole sample (*n* = 84)	Low SES group (*n* = 43)	High SES group (*n* = 41)
Child gender, *n* (%) male^a^	46 (55)	27 (63)	19 (46)
Child age, mean (SD) months^b^	41 (3)	41 (3)	42 (4)
Child ethnicity, *n* (%) White British^c^	78 (93)	37 (86)	41 (100)*
Household composition, *n* (%) live with both parents^a^	72 (86)	31 (72)	41 (100)***
Birth order, *n* (%) first born^a^	41 (49)	18 (42)	23 (56)
Maternal age at child's birth, mean (SD) years^a^	28 (6)	25 (5)	31 (5)***

a Comparison between socioeconomic groups by chi-square test.

b Comparison between socioeconomic groups by independent samples *t*-test.

c Comparison between socioeconomic groups by Fisher's exact test.

* *p* ≤ .05.

** *p* ≤ .01.

*** *p* ≤ .001.

Parents of 36 children were invited to participate in actigraphy, and 35 consented (97% consent rate). Eighteen children completed the full five days/nights and were included in analyses (12 from the high SES and six from the low SES group). Parents of the other 17 children reported that their children refused to keep the actiwatch on.

### Procedures

Ethical approval was granted by the Durham University Ethics Review committee. Between May 2008 and June 2009, parents were approached in person at nursery by C. J., a female social science researcher. They were informed about the study verbally and with a written information sheet. All participating parents, and representatives at each nursery, gave written informed consent.

We used a mixed methods approach, including parental interviews and parentally completed diaries for all participants, and actigraphy in a subgroup of children. Participating parents were interviewed by C. J. in a private room at their child's nursery. At the end of the interview, they were given a diary to complete and return the following week; some children also wore an actiwatch during this period. Interviews and diaries covered child sleep, diet and activity; only sleep data are presented here. Other aspects of the data are presented elsewhere (Jones & Ball, 2013; Jones, Pollard, Summerbell, & Ball, 2013).

Interviews lasted up to an hour and were digitally recorded. They were semi-structured, and the sleep section explored parents' descriptions of a typical bedtime for their child, including rules and routines regarding sleep, the reasons for using these or not, what they considered an appropriate sleep schedule and location for their child if any, and demographic information (interview schedule available on request). In keeping with the semi-structured design, all participants were prompted to discuss these topics, but answers to discrete questions were not required, so that participants could respond in their own terms. The interviewer ensured that the use or not of the sleep hygiene behaviours was discussed by all parents, either spontaneously or after specific prompting.

Diaries were completed during a typical week, for example, when children were not ill or on holiday, and we requested parents to document their child's bedtime, sleep onset time (the time at which they fell asleep), morning wake time, duration of any daytime naps and sleep onset location for five consecutive nights and the four intervening days (two week days and two weekend days).

A convenience subsample of children wore an actiwatch to coincide with the entire diary period, to validate parent-reported sleep–wake variables (Actiwatch Mini, Cambridge Neurotechnology Ltd). There was a limited number of actiwatches, and participants were invited to this section of the study according to whether a device was available at the time of the parents' interview. More actiwatches became available partway through the study, after data collection at some of the low SES nurseries had ceased and data collection at a high SES nursery was about to begin, meaning that more participants in the high SES group were invited. Actigraphy has been favourably validated against polysomnography for assessment of sleep in young children (Sadeh, Lavie, Scher, Tirosh, & Epstein, 1991; Shinkoda et al., 1998). Actigraphy data were downloaded and analysed in the proprietary software using the medium sensitivity setting, as recommended in the user manual.

### Data Analysis

For the qualitative analysis, interviews were listened to repeatedly by C. J., who identified themes regarding parents' reasons for using or not using sleep hygiene behaviours. These were discussed with H. B., and excerpts of interviews relevant to these themes were transcribed. For the quantitative analysis, interview data on sleep hygiene behaviours were systematically coded into categories by C. J. ‘Regular bedtime’ was coded if parents described implementing one on all nights, or most nights, for example, with flexibility on weekends; those who did not describe doing so were coded as ‘no regular bedtime’. ‘Reading at bedtime’ was coded if parents reported reading with/to their child during their typical bedtime routine; those who did not describe doing so were coded as ‘no reading at bedtime’. The coding scheme was blind-tested by an independent expert using a random sample of five audio-recoded interviews. Agreement on coding to these categories was 100% without the coders needing discussion, and so the coding method was considered to be reliable. Demographic information and usual sleep location (own room/shared room with own bed/shared bed) were also coded from the interview data. ‘Fell asleep in bed each night’ was coded if diaries indicated that children fell asleep for the night in bed every night, whether it was their own/their parents'/another bed. Those whose diary indicated that they fell asleep for the night on the sofa/in the living room on at least one night were coded as ‘did not fall asleep in bed each night’. Since coding for this behaviour was based on diary data rather than interview data, blind-testing for reliability of coding was not necessary.

For validation of parent-reported sleep–wake variables, diary-derived and actigraphy-derived sleep onset time, wake time and daytime nap duration were compared using paired samples *t*-tests and Pearson's correlations.

Nighttime sleep duration was calculated for each night for each participant using diary-reported sleep onset and wake times; duration of naps was totalled for each day. Weighted means for nighttime sleep duration and daily nap duration were calculated [(mean week night/day sleep duration × 5 + mean weekend night/day sleep duration × 2) ÷ 7]. These were totalled to obtain weighted mean total daily sleep duration per 24 h (nighttime plus naps) for each child. Weighted means were used because sleep duration has been found to vary between week and weekend nights in children as young as 3-years old (Snell, Adam, & Duncan, 2007). We have previously reported that sleep onset time and wake time were significantly later on weekend compared to week nights in this sample, whilst there were no significant differences in nighttime sleep duration or daily nap duration (Jones & Ball, 2013).

Nap duration was non-normally distributed, and nighttime and total sleep duration over 24 h were normally distributed; therefore median values are reported for nap duration, and mean values for nighttime and total daily sleep duration. Associations between variables were assessed with chi-square, Mann–Whitney *U*, Fisher's exact, and independent samples *t*-tests as appropriate. We considered associations to be statistically significant if *p* ≤ .05.

## RESULTS

### Validation of the Sleep Diary

Comparisons between actigraphy- and diary-derived sleep variables are shown in Table 2. The measures are closely correlated. The difference between diary- and actigraphy-derived mean sleep values varied from just 2 min for wake time to 8 min for sleep onset time. There were no systematic differences between families in the low SES and high SES groups (Jones, 2011). We therefore considered the diary a valid tool for assessing child sleep duration.

**Table 2:** Comparisons of diary- and actigraphy-derived sleep variables (subsample *n* = 18)

Sleep variable, hh : mm	Method (mean ± SD)		Paired samples *t*-test, *t*	Pearson's correlation, *r*
	Diary	Actigraphy		
Sleep onset time^a^	20:15 ± 1:00	20:23 ± 1:03	−1.52	.98***
Wake time^a^	07:31 ± 0:37	07:33 ± 0:38	−1.36	.99***
Nap duration^b^	0:12 ± 0:25	0:17 ± 0:14	−1.1	.81***

a Mean of 5 nights.

b Mean of 4 days.

*** *p* ≤ .001.

### Children's Sleep Duration and Sleep Hygiene Behaviours

Diary-derived sleep duration is shown in Table 3. Mean nighttime sleep duration for the sample was 11:12 ± 0:38 h, and mean total daily sleep duration including naps was 11:24 ± 0:35 h. For 80% of children, sleep duration over 24 h fell within the US National Sleep Foundation's recommended range for preschool children of 11–13 h per day; for 20% of children, sleep duration fell below this range. Half the children (51%) experienced at least one daytime nap during the diary period. Median daily nap duration for all children was just 1 min.

**Table 3:** Children's sleep duration

Sleep duration variable, hh : mm (weighted means)	Whole sample	Low SES group	High SES group
Nighttime sleep duration, mean (SD)^a^	11:12 (0:38)	11:09 (0:36)	11:16 (0:40)
Daytime sleep duration, median (interquartile range)^b^	0:01 (0:21)	0:09 (0:29)	0:00 (0:08)**
Total sleep duration over 24 h, mean (SD)^a^	11:24 (0:35)	11:26 (0:30)	11:22 (0:40)

a Comparison between socioeconomic groups by independent samples *t*-test.

b Comparison between socioeconomic groups by Mann–Whitney *U* test.

** *p* ≤ .01.

No difference was found in nighttime sleep or total daily sleep duration over 24 h between the low SES and high SES groups; however daily nap duration was significantly longer in the low SES group (median 9 min compared to 0 min) (see Table 3).

Around two-thirds of children slept in their own bedroom (69%); 23 children (27%) shared a room with a relative but had their own bed, and three children (4%) shared a bed with their parent or sibling. The prevalence of sleep hygiene behaviours is shown in Table 4. Two-thirds of parents reported reading at bedtime (63%), and more implemented a regular bedtime for their child (79%); 77% of children fell asleep in bed every night. There were significant associations between each of these behaviours (regular bedtime and reading at bedtime, *X*^2^ = 8.72, *p* = .003; regular bedtime and fell asleep in bed each night, Fisher's exact test *p* ≤ .001; reading at bedtime and fell asleep in bed each night, *X*^2^ = 4.65, *p* = .03).

**Table 4:** Associations between sleep hygiene behaviours and socioeconomic group

Sleep hygiene behaviour	Whole sample, *n* (%)	Low SES group, *n* (%)	High SES group, *n* (%)
Regular bedtime	66 (79)	30 (70)	36 (88)*
Reading at bedtime	53 (63)	18 (42)	35 (85)***
Fell asleep in bed each night	65 (77)	28 (65)	37 (90)*

All comparisons between socioeconomic groups by chi-square tests.

* *p* ≤ .05.

*** *p* ≤ .001.

### Parents' Reasons for Using or Not Using Sleep Hygiene Behaviours

Parents who set regular bedtimes spontaneously reported various reasons for doing so; most commonly so that children get enough sleep, so that parents have their own free time in the evening when their children are in bed, and so that children know what to expect, which prevents bedtime resistance: ‘she knows it's time to go to bed, and goes to sleep’ (parent in the high SES group, regular bedtime). Parents also frequently mentioned that regular bedtimes help children to feel secure and happy: ‘I think it's comfort, he seems to enjoy it’ (parent in the low SES group, regular bedtime). Reasons for not setting regular bedtimes included parents thinking that they were unnecessary or feeling unable to implement to them: ‘A kid'll know when they're tired, when they're tired they'll go to sleep’ (parent in the low SES group, no regular bedtime). A small number of parents disliked the impact that a regular bedtime would have on the whole family: ‘I don't think my life or my husband's life should be dictated by children's routine, if we want to do something we do and he comes along and goes to bed when he gets in’ (parent in the high SES group, no regular bedtime).

Reading at bedtime was commonly perceived to be enjoyable for both parents and children, and a component of quality family time. It was sometimes used as a treat or reward: ‘If she's been good in the day I might read her more’ (parent in the low SES group, reads at bedtime). A small number of parents negatively perceived the time and effort required, which prevented them from regularly reading to their children at bedtime.

The majority of parents intended their children to fall asleep in bed each night. No parents described the intention for their children to fall asleep on the sofa at night, despite the fact that almost a quarter of children did so on at least one of the five diary nights. When this did happen, it was sometimes driven by child reluctance to go to bed and parental difficulty taking them: ‘I can see she is tired and suggest taking her to bed, she says she wants to fall asleep on the sofa’ (parent in the low SES group, did not fall asleep in bed each night). Other parents did not take their children to bed before they fell asleep because they were not aware that they were falling asleep, or were unable or reluctant to take them to bed at the appropriate time: ‘can't be bothered taking him, he may as well sleep on the sofa’ (parent in the low SES group, did not fall asleep in bed each night).

Associations between sleep hygiene behaviours and socioeconomic status are shown in Table 4. Each of the sleep hygiene behaviours was significantly more prevalent in the high SES compared to the low SES group. The biggest difference was in reading at bedtime (85% in the high SES compared to 42% in the low SES group).

### Associations between Sleep Hygiene Behaviours and Sleep Duration

Associations of sleep hygiene behaviours with sleep duration are shown in Table 5. Children who had a regular bedtime, read at bedtime or fell asleep in bed every night experienced significantly longer nighttime sleep compared to their counterparts: the difference in mean sleep duration was 26, 22 and 25 min per night for each behaviour, respectively. There were no significant associations with total sleep duration over 24 h. Children who began their nighttime sleep on the sofa at least one night exhibited significantly longer daytime napping than those who fell asleep in bed every night: the difference in mean nap duration was 25 min per day.

**Table 5:** Associations between sleep hygiene behaviours and sleep duration

		Sleep duration variable (weighted means) (hh : mm)		
		Nighttime sleep duration	Daytime sleep duration (naps)	Sleep duration over 24 h
Sleep hygiene behaviour		Mean (SD)	Median (interquartile range)	Mean (SD)
Regular bedtime	Yes	11:18 (0:33)	0:01 (0:15)	11:27 (0:33)
	No	10:52 (0:49)**	0:06 (0:33)^b^	11:13 (0:41)^a^
Read at bedtime	Yes	11:20 (0:37)	0:00 (0:09)	11:28 (0:37)
	No	10:58 (0:36)**	0:04 (0:30)^b^	11:18 (0:31)^a^
Fell asleep in bed each night	Yes	11:18 (0:36)	0:00 (0:09)	11:25 (0:37)
	No	10:53 (0:39)**	0:25 (0:34)***	11:22 (0:28)^a^

a Independent samples *t*-test.

b Mann–Whitney *U* test.

** *p* ≤ .01.

*** *p* ≤ .001.

## DISCUSSION

### Main Findings

In this sample of preschool children from a socioeconomically diverse town in North-East England, there was great variability in parents' attitudes towards and use of recommended sleep hygiene behaviours (regular bedtime, reading at bedtime, falling asleep in bed each night). Parents who implemented sleep hygiene behaviours described, without specific prompting, various reasons for doing so including children feeling secure and happy, being able to achieve sufficient sleep and the mutual enjoyment of quality time together. Some parents who did not implement sleep hygiene behaviours did not feel they were necessary, perceiving sleep to be driven by children themselves rather than parents. Additionally, a number of reasons for not using sleep hygiene behaviours related to parental difficulty, inability or inconvenience in doing so, for example, the time required to read, and the time and effort required to take children to bed at a regular time when awake, particularly when they resisted. Sleep hygiene behaviours were more common in families in the high SES compared to low SES group.

Sleep hygiene behaviours were associated with significantly longer nighttime sleep, but not total sleep duration over 24 h. Mean total daily sleep duration was within the age-related recommendation of 11–13 h regardless of whether parents implemented each of the behaviours (see Table 5). The pattern of results suggests that longer daytime napping compensated for shorter nighttime sleep among children whose parents did not implement sleep hygiene behaviours, resulting in similar and sufficient sleep duration in the groups of children whose parents did versus did not implement sleep hygiene. However, evidence regarding the importance of sleep composition, i.e. nighttime sleep only compared to combined daytime and nighttime sleep, for sleep-related health is mixed (see Implications section below).

### Comparisons with Existing Literature

Mean total sleep duration across the sample (11:24) closely matches that of 3-year-old children in a large English cohort (11:31) (Blair et al., 2012). Parents implementing one sleep hygiene behaviour were significantly more likely to implement the others, as reported previously (Owens et al., 2011). This may reflect distinct parental approaches towards children's sleep – a structured approach versus an unstructured approach – and supports the hypothesis that families who read at bedtime provide more structure, which could positively impact sleep (Mindell et al., 2009). Both approaches were associated with similar, sufficient daily sleep amounts.

Our study provides further evidence that the implementation of sleep hygiene behaviours in healthy, non-sleep-disordered children is associated with longer nighttime sleep, and that this association holds true for British and socioeconomically diverse children. Our novel contribution is the exploration of why parents do or do not use sleep hygiene behaviours, and the finding that shorter nighttime sleep associated with lack of sleep hygiene is compensated for by increased daytime sleep. One other study examined combined daytime and nighttime sleep and found, unlike our results, that children with regular bedtimes were more likely to obtain sufficient sleep than those without (Owens et al., 2011). However, other practices discouraged by sleep hygiene, such as parental presence at sleep onset and having a television in the bedroom, were not associated with sleep sufficiency, which supports our findings. Differences between Owens et al.'s study and the present study include location (USA), a wide age range (3 months to 12 years), the use of cut-offs for sleep sufficiency rather than sleep duration as a continuous variable, and a socioeconomically homogenous sample comprised of largely college-educated, higher socioeconomic status participants.

Sleep hygiene behaviours were more prevalent in the high SES group, consistent with previous reports (Hale et al., 2009); but there were no significant differences in nighttime sleep or sleep duration over 24 h between socioeconomic groups. In their cohort of over 11,000 British children, Blair and colleagues (2012) found that total daily sleep (nighttime plus naps) was shorter amongst boys compared to girls, children with older compared to younger mothers, non-White compared to White ethnicity children, and children with more siblings. However, total daily sleep duration was not associated with markers of socioeconomic status in our or Blair's study. This is surprising given that sleep hygiene behaviours and bedtime routines, which are associated with sleep duration, are less common in households of lower socioeconomic status (Blair et al., 2012; Hale et al., 2009). We suggest this anomaly could be due to variation in daytime napping between different socioeconomic groups compensating for differences in nighttime sleep.

### Implications

Our results provide insight into how sleep hygiene may be promoted amongst those (particularly low SES) families who do not employ sleep hygiene practices. Parents may need to be ‘persuaded’ that parental input is needed to help control children's sleep and that sleep should not be driven by children alone. Furthermore, practical advice on how to implement these behaviours, for example, how to manage bedtime resistance, may be needed, because the effort and time required, particularly for reluctant children, were reasons for not employing these behaviours. An educational intervention was found to be successful at increasing parents' knowledge regarding children's healthy sleep and resulted in an increase in the number of parents planning to make positive changes to their child's sleep practices (Jones, Owens, & Pham, 2012). However, simply educating parents on the benefits of sleep hygiene may not result in intention or ability to implement these behaviours if the barriers identified here are not addressed.

Importantly, though, our results question the assumption that sleep hygiene behaviours should be promoted amongst all families. Children whose parents did not implement sleep hygiene did not have significantly shorter sleep over a 24-h period compared to those whose parents did. Further research is needed to determine whether composition of daily sleep (nighttime only versus combined nighttime plus naps) is important for children's health and development, as there is currently a lack of consensus. Whilst nighttime sleep is important for biological, psychosocial and restorative functions, daytime sleep can positively impact attention span and alertness as well as reduce psychosocial stress (Bell & Zimmerman, 2010; Ward, Gay, Alkon, Anders, & Lee, 2008). A recent study showed that daytime naps in preschool children enhance memories and support learning (Kurdziel, Duclos, & Spencer, 2013). There is a well-documented association between short sleep duration and obesity in young children (Chen et al., 2008), and one study found that the 30-min difference in sleep duration at ages 3–5 years, associated with obesity at age 9.5 years, was almost entirely due to napping (Agras, Hammer, McNicholas, & Kraemer, 2004). However, higher proportion of total sleep occurring at night has been positively associated with executive functioning in infants (Bernier, Carlson, Bordeleau, & Carrier, 2010), and daytime napping was negatively correlated with neurocognitive function in pre-schoolers (Lam, Mahone, Mason, & Scharf, 2011). Although these findings indicate that cessation of napping is a marker for brain development, we have previously reported that in our sample of children, positive parental attitude towards napping was associated with longer child nap duration, suggesting that napping may be influenced by parental attitudes as well as being biologically determined (Jones & Ball, 2013). Determining the importance of sleep composition would reveal whether longer nighttime sleep (which is associated with sleep hygiene behaviours) is more optimal than shorter nighttime sleep compensated with daytime napping (which is associated with lack of sleep hygiene behaviours).

There is a focus in the literature on primarily middle-class US parenting approaches, involving use of sleep hygiene behaviours, which indeed are associated with longer sleep in that group. It is widely known that national polls and internet-based surveys are skewed towards well-educated, middle-class respondents, for example, education level in two large cohorts was greater than the average of the populations from which they were taken (Mindell et al., 2010; Sadeh et al., 2009). Sleep patterns derived from these samples will represent the normative sleep of children whose parents have certain expectations and practices, i.e. who likely implement sleep hygiene, resulting in these behaviours being accepted as the norm or optimal way for children to sleep. Furthermore, there has been a focus on the association of sleep hygiene behaviours with nighttime sleep, which our study suggests neglects the compensation for short nighttime sleep with daytime napping in those children whose parents do not implement sleep hygiene. Families utilizing an unstructured approach to children's sleep, who are predominantly of lower socioeconomic status, and who compensate for short nighttime sleep with longer daytime napping, are not fully represented in survey-based estimates of normative sleep amounts.

This anthropological study has revealed the diversity in what parents perceive to be appropriate or acceptable ways to manage their children's sleep. We encourage others to acknowledge and seek to understand the diversity of parenting approaches and child sleep patterns.

### Strengths and Limitations

This study is an important and novel addition to the literature regarding children's sleep, providing much-needed data on British preschool children's sleep. Importantly, we recruited a socioeconomically diverse sample, addressing the lack of data on sleep in less affluent populations. Furthermore, the mixed methods design allowed us to explore why parents practice sleep hygiene behaviours or not, rather than looking at prevalence of sleep hygiene alone, which gives us insight into the context of these behaviours, and could inform how to promote sleep hygiene more effectively if this is appropriate.

We note a number of limitations to our study. Sleep was assessed by parental report, which is naturally subjective, and did not assess time spent awake during the night. It is common practice in the literature to estimate sleep duration from parents' estimates of usual bedtime, wake time and nap duration, or usual sleep duration and to not include night wakings in this estimate, for example (Iglowstein et al., 2003; Owens et al., 2011). Hence despite limitations, our sleep duration estimate was relatively robust due to averages calculated over 5 nights/4 days, and validation against actigraphy.

Sleep quality was not assessed in our study: some children may have experienced poorer sleep quality and/or spent longer periods of the night awake. This is important because it is argued that sleep hygiene behaviours promote improved sleep quality in addition to duration, which is positively associated with health and development (Galland & Mitchell, 2010). Future research should examine whether sleep quality differs between children who have similar sleep durations but different use of sleep hygiene behaviours. Other sleep hygiene behaviours, which may be associated with sleep duration, were not investigated (including regular wake time, absence of caffeine consumption and bedroom electronics), and we did not investigate co-sleeping or children's sleep location through the night, for example, whether children moved locations through the night after falling asleep. However, the sleep hygiene behaviours we selected have previously been shown to be associated with young children's sleep duration and are commonly recommended. This was a purposive sample involving 84 families, and caution should be taken in applying the results to other populations of preschool children. However, generalizability was improved by including a socioeconomically diverse sample, with response and participation rates being similar between the low and high SES groups. Parents' reasons for employing or not employing sleep hygiene behaviours are likely to be context specific, but the finding that daytime napping compensates for shorter nighttime sleep in children without sleep hygiene may be more generalizable and should be examined in future studies. One other aspect our data does not address is sleep pressure, for example, do children with shorter nighttime sleep and lack of sleep hygiene build up sleep pressure, which results in opportunistic daytime sleep to catch up, or do children who nap then not need as much sleep at night and therefore not benefit from practices which promote longer nighttime sleep.

## CONCLUSION

This study confirms previous reports that sleep hygiene behaviours are associated with longer children's nighttime sleep and extends these findings to healthy British preschool children. Importantly, we found that longer daytime napping compensated for shorter nighttime sleep, resulting in similar overall sleep amounts for children whose parents did and did not implement sleep hygiene behaviours. Children in the high SES group were more likely to experience sleep hygiene behaviours than those in the low SES group, but they did not have longer sleep overall. Further research is needed to examine whether composition of daily sleep is important for children's health and behaviour. This study also describes reasons why parents do or do not practice sleep hygiene. We encourage the sleep medicine community to consider the contexts in which sleep hygiene behaviours are practiced or not practiced, and consider how to promote optimal sleep practices for families with different expectations and attitudes.
